# Climate Change and Eutrophication Induced Shifts in Northern Summer Plankton Communities

**DOI:** 10.1371/journal.pone.0066475

**Published:** 2013-06-12

**Authors:** Sanna Suikkanen, Silvia Pulina, Jonna Engström-Öst, Maiju Lehtiniemi, Sirpa Lehtinen, Andreas Brutemark

**Affiliations:** 1 Marine Research Centre, Finnish Environment Institute, Helsinki, Finland; 2 Department of Science for Nature and Environmental Resources, University of Sassari, Sassari, Italy; 3 ARONIA Coastal Zone Research Team, Novia University of Applied Sciences and Åbo Akademi University, Ekenäs, Finland; 4 Tvärminne Zoological Station, Hanko, Finland; University of Connecticut, United States of America

## Abstract

Marine ecosystems are undergoing substantial changes due to human-induced pressures. Analysis of long-term data series is a valuable tool for understanding naturally and anthropogenically induced changes in plankton communities. In the present study, seasonal monitoring data were collected in three sub-basins of the northern Baltic Sea between 1979 and 2011 and statistically analysed for trends and interactions between surface water hydrography, inorganic nutrient concentrations and phyto- and zooplankton community composition. The most conspicuous hydrographic change was a significant increase in late summer surface water temperatures over the study period. In addition, salinity decreased and dissolved inorganic nutrient concentrations increased in some basins. Based on redundancy analysis (RDA), warming was the key environmental factor explaining the observed changes in plankton communities: the general increase in total phytoplankton biomass, Cyanophyceae, Prymnesiophyceae and Chrysophyceae, and decrease in Cryptophyceae throughout the study area, as well as increase in rotifers and decrease in total zooplankton, cladoceran and copepod abundances in some basins. We conclude that the plankton communities in the Baltic Sea have shifted towards a food web structure with smaller sized organisms, leading to decreased energy available for grazing zooplankton and planktivorous fish. The shift is most probably due to complex interactions between warming, eutrophication and increased top-down pressure due to overexploitation of resources, and the resulting trophic cascades.

## Introduction

Environmental monitoring data are being collected worldwide to assess the current state of marine ecosystems, as well as to identify spatial and temporal changes associated with eutrophication, climate or other human induced pressures across the world's oceans [1], [2]. Long-term data series are a prerequisite for understanding the changes and for separating natural inter-annual variation from anthropogenic influence in hydrography, plankton abundance and biodiversity.

The cumulative effects of global change, including climate warming and increased human population, followed by more intense industrialisation and agribusiness, will probably continue and intensify the course of eutrophication in estuarine waters. Global climate change will likely result in higher water temperatures, stronger stratification and increased inflows of freshwater and nutrients to coastal waters in many areas [3].

Phytoplankton form the basis for aquatic food webs as they constitute the largest photosynthesising biomass on earth, passing both coral reefs and rainforests in oxygen production [4]. In nutrient-poor and high latitude waters, the spring bloom is the single seasonal peak of primary production, providing energy and matter base for zooplankton, benthic animals and fish [5]. In nutrient-rich areas, late-summer blooms consisting of harmful phytoplankton species often occur [6]. Zooplankton are the most important secondary producers in oceans and they represent the interface between primary producers and planktivores, and thereby form the crucial link for the energy transfer to higher trophic levels, i.e. basically all fish larvae, as well as many commercially important planktivorous fish [7]. Large changes in plankton abundances may thus have serious effects on ecosystem functioning via bottom-up and top-down effects cascading through the food webs [8], [9].

Recently, considerable changes in plankton phenology, distribution, species composition and abundance have been detected. Some examples from phytoplankton include a decreased diatom : dinoflagellate ratio in the Baltic Sea spring bloom [10] and, in the central North Sea, changed phenology, indicating changes in initiation or peak of the phytoplankton bloom [11]. The risk of harmful phytoplankton blooms in the future has increased due to climate change and eutrophication interactions [2], [6]. For zooplankton, different phenomena are observed, such as increases in the proportion of small-sized species and young age classes, and decreases in size-at-age [12]. Other examples include higher species turn-over in coastal ecosystems due to warming [13], temporal mismatch between predators and their prey [14], and negative correlations between copepod populations (*Calanus*) and the NAO index [15].

Richardson et al. [16] showed that there is a serious knowledge gap of climate change effects on the marine ecosystem, and that considerably more commitment should be given to time series. To date, comprehensive studies on long-term changes in the Baltic Sea have not been conducted by analysing both phyto- and zooplankton class-level community data (but see e.g. [8], [17], [18] for recent analyses of Baltic Sea monitoring data). Based on statistical analysis of existing monitoring data collected in three sub-basins of the Baltic Sea for more than 30 years, we demonstrate strong effects by climate change and eutrophication on hydrography and plankton communities, causing a shift towards a food web structure composed of low-food-quality phytoplankton and small sized zooplankton species leading to decreased energy availability for predators.

## Materials and Methods

### Ethics statement

No permits were required for the described study, and the study did not involve endangered or protected species.

### Study area

**Figure 1:**
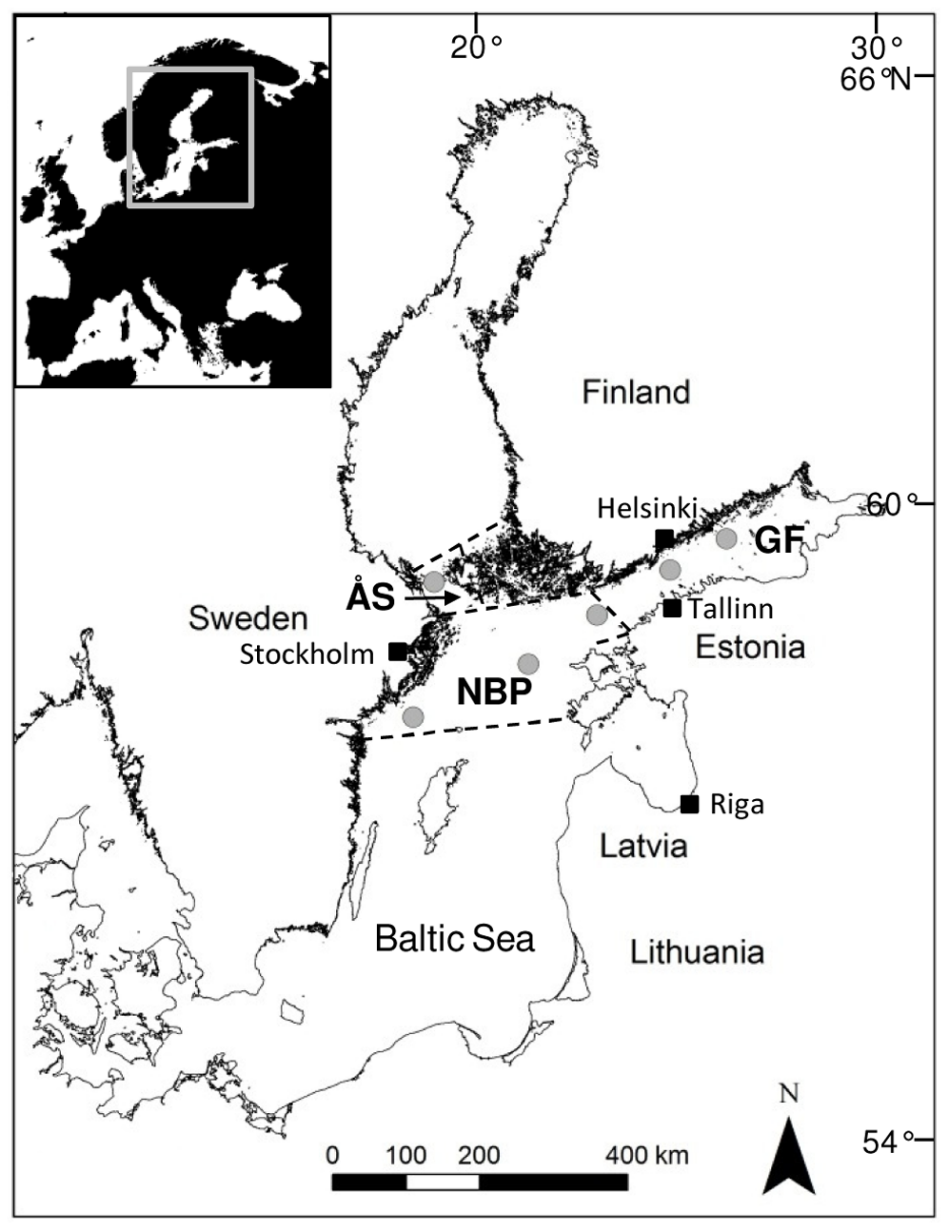
Location of the monitoring stations. The three sub-basins of the Baltic Åland Sea (ÅS), Gulf of Finland (GF) and northern Baltic proper (NBP) are bordered with dashed lines.

The Baltic Sea is a shallow semi-enclosed brackish water basin (ca. 422 000 km^2^, mean depth 55 m), with a restricted connection to the North Sea via the Danish straits (Fig. 1). Functionally the Baltic Sea is a large estuary with both horizontal and vertical salinity gradients, and a deep and permanent halocline at a depth of ca. 60–80 m in the Baltic proper and the Gulf of Finland. Also, a summer thermocline lies at a depth of ca. 10–20 m. Due to the partly enclosed geography, the slow water renewal time and the high anthropogenic nutrient load from the land, the Baltic Sea is highly at risk for eutrophication [17]. In addition, this ecosystem is sensitive to climate change, mainly because it is greatly affected by freshwater runoff, and by saltwater intrusions from the North Sea, which are forced by meteorological conditions [19]. The Baltic consists of several sub-basins, each with its own characteristic properties. Our data originate from six sampling stations located in three sub-basins: three stations in the northern Baltic proper (NBP), two in the Gulf of Finland (GF) and one in the Åland Sea (ÅS; Fig. 1). The NBP (29100 km^2^) and GF (29500 km^2^), which are connected without a sill, cover an area much larger than that of the ÅS (8000 km^2^), which is separated from the NBP by a sill. GF is located between Finland and Estonia and is considered as one of the most eutrophicated sub-basins of the Baltic Sea [20]. Oxygen deficient near-bottom areas connected with internal nutrient loading are common in the GF and NBP, but not in the ÅS.

### Data

Our time series data from 1979 to 2011 originate from national monitoring cruises, where sampling is conducted according to the Helsinki Commission (HELCOM) COMBINE programme. The data were downloaded from the marine monitoring database Sumppu of the Finnish Environment Institute and the Finnish Meteorological Institute (nodc.fmi.fi/grafeio), and from the national Hertta database (wwwp2.ymparisto.fi/scripts/oiva.asp), which is run by the Finnish Environment Institute. For all analyses involving phytoplankton, data was available from 1979 to 2008 only.

Late summer data of hydrographic variables (temperature, salinity and pH), chlorophyll *a* concentration, phytoplankton biomass and zooplankton abundance were used (Table 1). There were usually 1–3 samplings per sea area per year. Zooplankton abundance was used instead of biomass due to an ongoing revision of zooplankton biomass calculations that concern the Baltic Sea area. For the concentrations of dissolved inorganic nutrients (nitrogen, phosphorus and silicate), measurements at the same stations from the preceding winter were considered. Long-term trends in surface water nutrient concentrations are most reliably detected in winter samples, and winter nutrient trends can be expected to be linked to summer plankton communities over the long term [21].

**Table 1:** Sampling and analysis methods of the environmental and plankton variables.

Variable	Data period	Depth	Sampling method	Analytical method
Temperature, salinity, pH	29 July - 3 Sept 1979–2011	Mean 0–10 m	SBE 911plus CTD system and Rosette sampler	pH determination based on standard SFS 3021 (1979): pH-meter connected with a glass/reference electrode
Dissolved inorganic nutrients (DIN, DIP, SiO_4_)	1 Nov – 31 Jan 1978–2011	Mean 0–10 m	Rosette sampler	Spectrophotometric measurement [50], [51]; detection limit 0.01 µM
Chlorophyll *a*	29 July - 3 Sept 1979–2011	Mean 0–10 m	Rosette sampler	Spectrofluorometric measurement [52], [53]
Phytoplankton biomass	29 July - 3 Sept 1979–2008	Integrated sample from 0–10 m	Rosette sampler, fixation with Lugol	Counting with inverted microscope [54], biomass calculation [53], [55]
Zooplankton abundance	29 July - 3 Sept 1979–2011	0–25 m (1979–1988), 0-thermocline (1989–2011)	Vertical tows of WP2 net (mesh 100 µm) with a flow meter, fixation with formaldehyde	Counting with inverted microscope to the lowest taxonomic level possible [53]

### Statistical analyses

**Figure 2:**
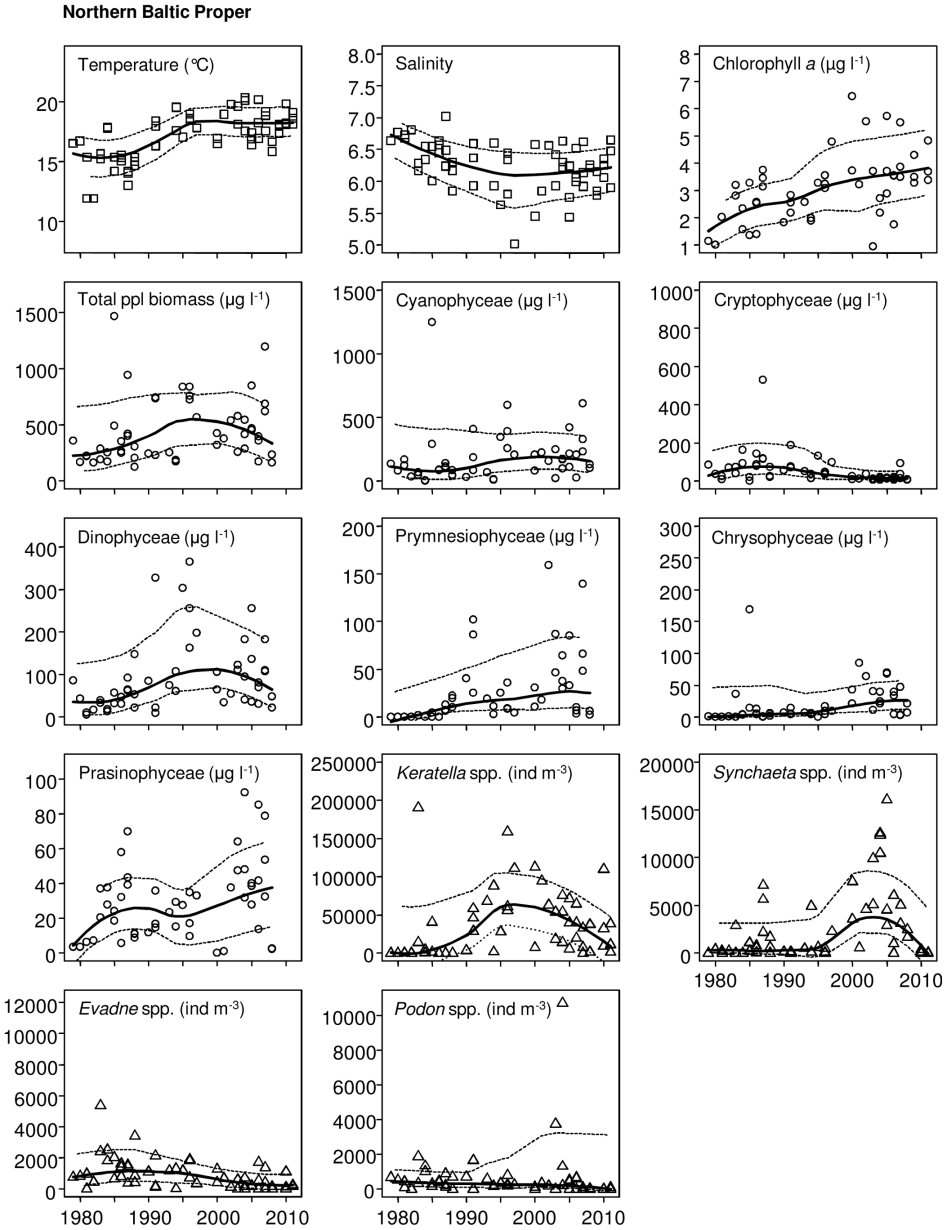
Significant trends (*p*<0.05) in environmental (squares) and zooplankton parameters (triangles) in the northern Baltic proper from 1979 to 2011, and in phytoplankton (circles) from 1979 to 2008. A Loess curve (solid line) is fitted, with a 95% confidence interval (dashed line).

**Figure 3:**
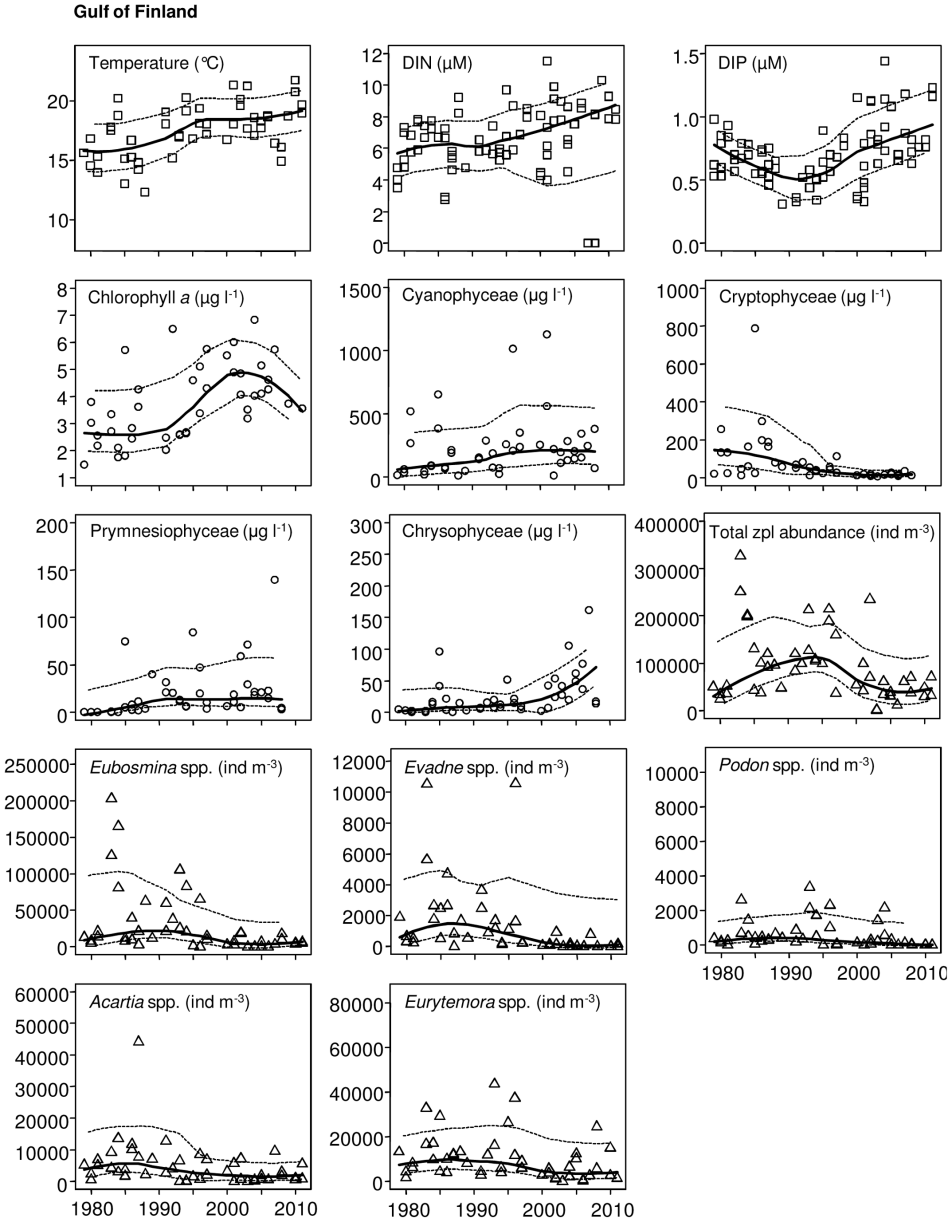
Significant trends (*p*<0.05) in environmental (squares) and zooplankton parameters (triangles) in the Gulf of Finland from 1979 to 2011, and in phytoplankton (circles) from 1979 to 2008. A Loess curve (solid line) is fitted, with a 95% confidence interval (dashed line).

**Figure 4:**
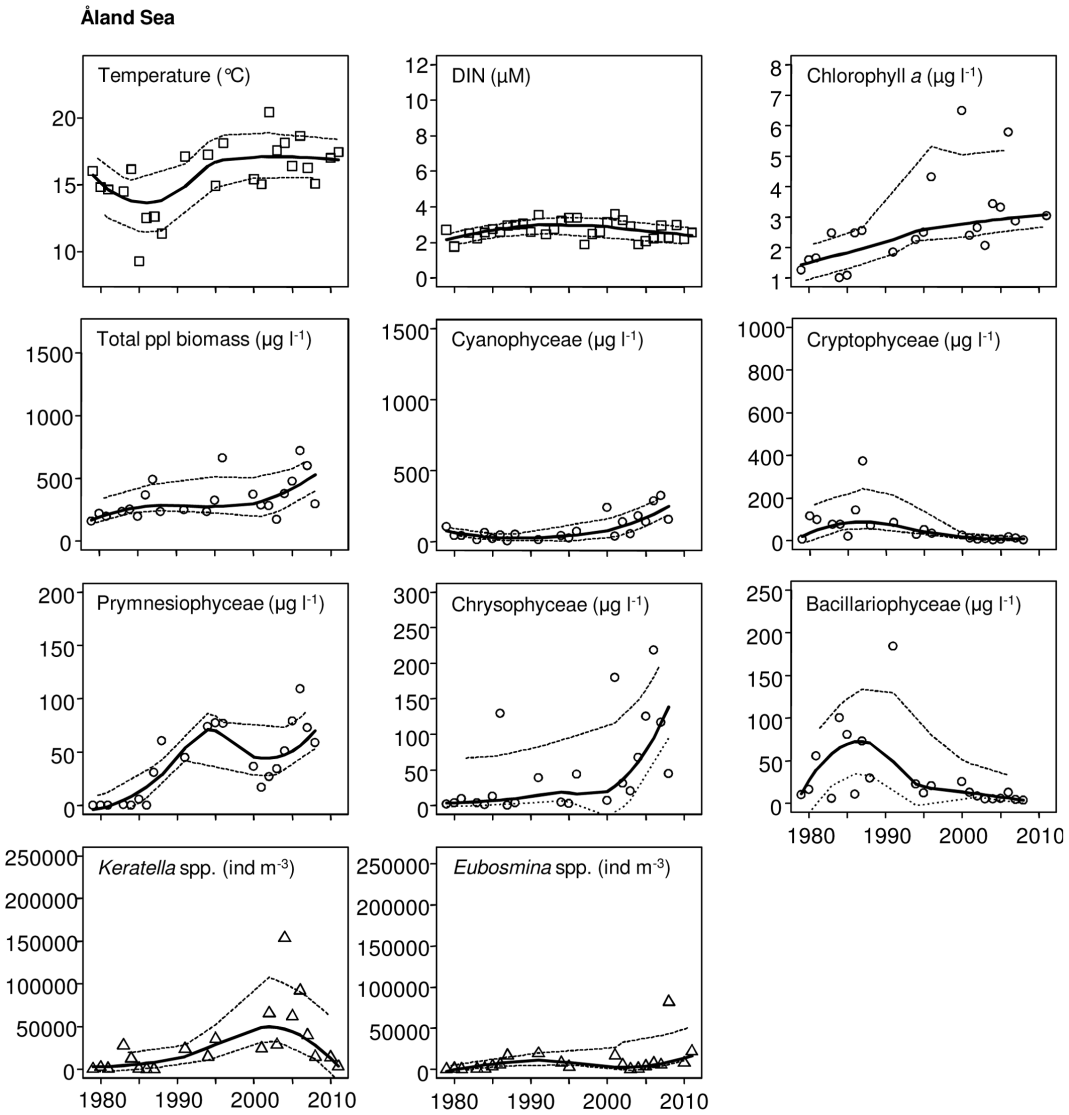
Significant trends (*p*<0.05) in environmental (squares) and zooplankton parameters (triangles) in the Åland Sea from 1979 to 2011, and in phytoplankton (circles) from 1979 to 2008. A Loess curve (solid line) is fitted, with a 95% confidence interval (dashed line).

The non-parametric Mann-Kendall test was used to detect significant monotonic trends in phytoplankton, zooplankton and environmental time series data. Curves estimated with a locally weighted scatterplot smoother (LOESS; span  =  0.75) with 95% confidence interval were fitted to describe the long-term variation (Figs. 2, 3, 4). Only statistically significant trends (*p*<0.05) are shown in Figures 2, 3, 4.

Redundancy analysis (RDA) was used to assess relationships between the plankton community composition and environmental variables (temperature, salinity and concentrations of DIN, DIP and SiO_4_) [22]. The nine phytoplankton classes and seven zooplankton genera included in our analyses are the most abundant and were identified consistently in all three targeted sub-basins throughout the analysis period (1979–2008). Phytoplankton biomass data and zooplankton abundance data were log(x+1)-transformed to stabilize variance and reduce the influence of dominant taxa on the ordination.

All statistical analyses were performed in R 2.15.2 [23] and tests were considered significant when the p-value was less than 0.05.

## Results

### Trends in hydrography

Temperature showed a highly positive significant trend in all studied sub-basins: the NBP, GF and ÅS (Table 2; Figs. 2, 3, 4). Salinity, on the other hand, decreased significantly only in the NBP (Table 2; Fig. 2). Wintertime dissolved inorganic nitrogen (DIN) showed a significant increase in the GF and ÅS, whereas dissolved inorganic phosphorus (DIP) increased only in the GF (Table 2; Figs. 3–4). pH, DIN:DIP ratio and silicate (SiO_4_) showed no significant changes in the study area during the time period 1979–2011 (Table 2).

**Table 2:** Results of the Mann-Kendall test for detection of long-term trends.

	Northern Baltic proper			Gulf of Finland			Åland Sea		
	*S*	*p*	*n*	*S*	*p*	*n*	*S*	*p*	*n*
Temperature	**704**	**<0.001**	**59**	**383**	**<0.001**	**49**	**106**	**0.009**	**24**
Salinity	**–436**	**0.004**	**59**	–130	0.266	49	–51	0.214	24
pH	115	0.432	57	85	0.441	47	51	0.158	23
DIN	–61	0.770	72	**549**	**0.012**	**75**	**163**	**0.008**	**32**
DIP	–62	0.767	72	**549**	**0.012**	**75**	17	0.795	32
DIN:DIP	25	0.907	72	26	0.909	75	117	0.057	32
SiO_4_	89	0.669	72	**–**46	0.837	75	96	0.123	32
Chlorophyll *a*	**477**	**<0.001**	**50**	**357**	**<0.001**	**43**	**113**	**<0.001**	**22**
Total phytopl. biomass	**270**	**0.034**	**52**	104	0.314	45	**109**	**0.002**	**22**
Cyanophyceae	**296**	**0.020**	**52**	**220**	**0.032**	**45**	**103**	**0.004**	**22**
Cryptophyceae	**–471**	**<0.001**	**52**	**–467**	**<0.001**	**45**	**–127**	**<0.001**	**22**
Dinophyceae	**397**	**0.002**	**52**	–68	0.512	45	17	0.652	22
Prymnesiophyceae	**616**	**<0.001**	**52**	**401**	**<0.001**	**45**	**129**	**<0.001**	**22**
Chrysophyceae	**548**	**<0.001**	**52**	**444**	**<0.001**	**45**	**111**	**0.002**	**22**
Bacillariophyceae	–72	0.575	52	–185	0.072	45	**–99**	**0.006**	**22**
Euglenophyceae	–140	0.271	52	–19	0.857	45	65	0.061	22
Prasinophyceae	**301**	**0.018**	**52**	88	0.395	45	–13	0.735	22
Chlorophyceae	–110	0.390	52	173	0.092	45	–15	0.693	22
Total zoopl. abundance	101	0.502	58	**–266**	**0.022**	**49**	58	0.085	21
*Keratella* spp.	**380**	**0.011**	**58**	24	0.842	49	**75**	**0.025**	**21**
*Synchaeta* spp.	**299**	**0.045**	**58**	70	0.551	49	30	0.381	21
*Eubosmina* spp.	–278	0.063	58	**–424**	**<0.001**	**49**	**88**	**0.009**	**21**
*Evadne* spp.	**–594**	**<0.001**	**58**	**–614**	**<0.001**	**49**	**–**59	0.079	21
*Podon* spp.	**–454**	**0.002**	**58**	**–355**	**0.002**	**49**	6	0.880	21
*Acartia* spp.	–183	0.222	58	**–281**	**0.016**	**49**	–22	0.526	21
*Eurytemora* spp.	78	0.605	58	**–318**	**0.006**	**49**	8	0.833	21

Significant trends in the environmental factors, phytoplankton biomass and zooplankton abundance data (for cladocerans and copepods, sum of adults and juveniles or copepodites, respectively) are marked in bold. *S*  =  Kendall score, *p*  =  significance, *n*  =  number of observations.

### Phytoplankton and zooplankton trends

A significant increasing trend was observed for chlorophyll *a* in all three sub-basins during the 1979–2011 study period (Table 2; Figs. 2, 3, 4). The total phytoplankton biomass also increased between 1979 and 2008, but significantly only in the NBP and ÅS (Table 2; Figs. 2, 4). Among phytoplankton classes, significant increasing trends were recorded for Cyanophyceae, Prymnesiophyceae, and Chrysophyceae as well as a significant decreasing trend for Cryptophyceae in all sub-basins (Table 2; Figs. 2, 3, 4). Moreover, Dinophyceae and Prasinophyceae significantly increased in the NBP (Table 2; Fig. 2), and Bacillariophyceae decreased in ÅS (Table 2; Fig. 4). Dominating phytoplankton genera in the data set (defined by the mean biomass and frequency of occurrences) were the cyanobacteria *Aphanizomenon, Nodularia* and *Snowella*, the dinoflagellates cf. *Gymnodinium* and *Heterocapsa*, the haptophyte *Chrysochromulina* and the prasinophyte *Pyramimonas*.

The largest changes in zooplankton were observed in the GF where the total zooplankton abundance decreased significantly (Table 2). This decrease was mostly due to the very low total abundance during the last 10 years (Fig. 3). A significant decrease was also found in all dominant cladoceran and copepod genera including *Eubosmina, Evadne, Podon, Acartia* and *Eurytemora* (Table 2, Fig. 3). However, in *Eubosmina* and *Eurytemora* the decrease was significant only in adult stages, while no change was observed for juveniles (Table 3). *Evadne* and *Podon,* as well as adult *Acartia* and *Eubosmina* also decreased significantly in the NBP (Tables 2, 3; Fig. 2). The opposite was observed for the rotifers among which both dominating genera (*Keratella* and *Synchaeta*) increased significantly. Due to the opposite trends, the total zooplankton abundance in the NBP did not change. The zooplankton community in the ÅS changed the least of the study areas: *Keratella* and *Eubosmina* juveniles, only, increased significantly (Tables 2, 3; Fig. 4).

**Table 3:** Mann-Kendall trend test results for cladoceran and copepod abundance data divided into adults and juveniles or copepodites, respectively.

	Northern Baltic proper			Gulf of Finland			Åland Sea		
	*S*	*p*	*n*	*S*	*p*	*n*	*S*	*p*	*n*
*Eubosmina* adults	**–383**	**0.010**	**58**	**–490**	**<0.001**	**49**	63	0.061	21
juveniles	157	0.291	58	121	0.295	49	**115**	**<0.001**	**21**
*Evadne* adults	**–652**	**<0.001**	58						
* *juveniles	**301**	**0.009**	58						
*Acartia* adults	**–434**	**0.004**	**58**	**–314**	**0.007**	**49**	–50	0.139	21
* *copepodites	46	0.763	58	**–261**	**0.025**	**49**	–18	0.608	21
*Eurytemora* adults	–261	0.081	58	**–483**	**<0.001**	**49**	–23	0.506	21
* *copepodites	241	0.107	58	–190	0.103	49	12	0.740	21

(*S*  =  Kendall score, *p*  =  significance, *n*  =  number of observations). Data of *Podon* and *Evadne* spp. (GF, ÅS) were not tested due to small number of observations (<10) of juveniles. Significant trends are marked in bold.

### Relationships between plankton and environmental variables

**Figure 5:**
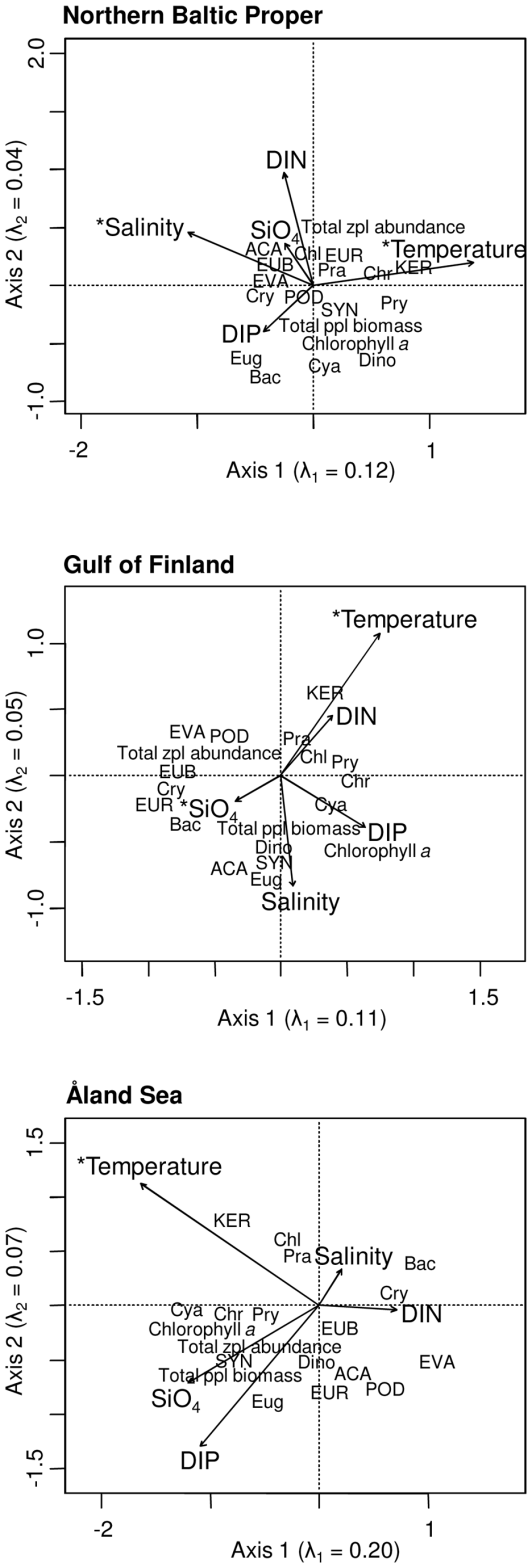
Correlation plots of the redundancy analysis (RDA) on the relationship between environmental parameters (vectors) and plankton variables during 1979–2008. Asterisks indicate statistical significance (*p*<0.05) of environmental variables. The plots display 15.5, 15.6 and 26.2% of the variance in the plankton data in the NBP, GF and ÅS, respectively, and eigenvalues of the first two axes are indicated by λ_1_ and λ_2_. Bac  =  Bacillariophyceae, Chl  =  Chlorophyceae, Chr  =  Chrysophyceae, Cry  =  Cryptophyceae, Cya  =  Cyanophyceae, Dino  =  Dinophyceae, Eug  =  Euglenophyceae, Pra  =  Prasinophyceae, Pry  =  Prymnesiophyceae, ACA  =  *Acartia* spp., EUB  =  *Eubosmina* spp., EUR  =  *Eurytemora* spp., EVA  =  *Evadne* spp., KER  =  *Keratella* spp., POD  =  *Podon* spp., SYN  =  *Synchaeta* spp.

The RDA results are displayed by an ordination plot for each sub-basin (Fig. 5). Together, all environmental variables accounted for 19.8%, 20.2% and 35.6% of the variation in the 1979–2008 plankton data in the NBP, GF and ÅS, respectively.

In all areas, temperature was the most significant environmental factor explaining variability in the plankton community (NBP: *F*  =  5.03, *p*<0.001, GF: *F*  =  3.20, *p*  =  0.001, ÅS: *F*  =  2.66, *p*  =  0.013). In addition, the effects of salinity in the NBP (*F*  =  2.38, *p*  =  0.011) and SiO_4_ in the GF were significant (*F*  =  3.02, *p*  =  0.003).

The plankton taxa were classified according to their association with the environmental variables (Fig. 5). Throughout the study area, both the total phytoplankton community (based on total phytoplankton biomass and chlorophyll *a* concentrations) and the phytoplankton classes that increased significantly in all areas (Cyanophyceae, Prymnesiophyceae and Chrysophyceae) were positively correlated with temperature and DIP concentration, but negatively with salinity and DIN concentration. High biomass of Cryptophyceae, the only phytoplankton class with significant decreasing trends in all areas, was consistently connected to low temperatures and high salinities. Among the rotifers that increased, the abundance of *Keratella* spp. was always positively related to temperature, but that of *Synchaeta* spp. was negatively associated with DIN concentrations. The abundance of the cladocerans (*Eubosmina*, *Evadne* and *Podon* spp.) and the copepods (*Acartia* and *Eurytemora* spp.), which decreased significantly in some sub-basins, generally correlated negatively with temperature.

## Discussion

Global warming has caused a remarkable rise in seawater temperatures worldwide [24] and similar increasing trends are detected in all our study areas. An interesting observation is that seawater warming in the studied basins was most intensive until the year 1995, but levelled out later (Figs. 2, 3, 4). Several reasons could explain this phenomenon; there may be slight bias caused by variation among sampling dates [21] or by physical processes that have affected surface temperature, caused by upwelling, mixing or other water movements [25].

Changes in climate patterns, such as increased precipitation, and related runoff regimes can significantly influence estuarine and coastal seawater salinity, as well as nutrient losses from catchments [19]. The estimated change in total mean annual river-flow to the Baltic Sea ranges from +2 to +15% of present-flow by year 2100, based on different scenarios [26]. Some scenarios forecast up to +30–40% increase in river flow [26]. Our long-term data analyses show that salinity is decreasing in the northern Baltic proper, and this result is supported by previous studies [21], [27]. In addition to an increased freshwater influence, another important factor is the decreasing inflow of salt water to the Baltic Sea via the Danish straits. The inflows have become increasingly rare and their volume smaller, which partly can be explained by the Northern Atlantic Oscillation (NAO) index that has been more positive than negative during the recent years [27]. Salinity is the main factor governing Baltic biodiversity [28] and its continuous decrease may have serious consequences for the plankton community composition.

In contrast to the consistent temperature increase in all study areas, trends in winter inorganic nutrient concentrations varied between the sub-basins. Increasing DIN and DIP concentrations in the GF, and DIN concentrations in the ÅS indicate an on-going eutrophication process in these areas. This result agrees with recent reports showing that nutrient concentrations increased in the Baltic Sea until 1980s, whereas DIP concentrations declined during the past two decades in all areas, except in the GF [20]. This can be clearly seen in the increasing chlorophyll *a* concentration in all study areas. Phytoplankton, as the primary producer, could be expected to respond first to eutrophication, but our results showed that the phytoplankton changes were similar in all study areas, despite differing nutrient trends. Increasing temperature thus seemed to have a greater effect on the phytoplankton community than eutrophication during the study period. Earlier studies have concluded that the long-term changes in the Baltic Sea phytoplankton communities probably reflect both climatic/hydrographic changes and the ongoing eutrophication process [21], [29]. The increase of the proportion of Dinophyceae during the spring bloom in many areas of the Baltic Sea has, however, been linked to weather and hydrographic conditions, not to changes in nutrient concentrations [10]. Olli et al. [30] also concluded, based on Baltic Sea-wide monitoring results from 1966 to 2008 that a continuous change is occurring in the Baltic Sea phytoplankton, even though the changes have only low associations with ambient nutrient concentrations and known eutrophication gradients during the period. Eutrophication and climatic/hydrographic changes are closely connected, since nutrient concentrations are not only affected by allochtonous nutrient loading, but also by climatic and hydrographic factors, such as mixing of the water column, which affects the oxygen conditions in the bottom sediments. The internal loading from anoxic bottom sediments is a major source of P in the GF, and thus strong mixing during autumn and late formation of ice cover can reduce P leaching from the sediments [20].

A closer look at the phytoplankton classes shows that the biomass of Cyanophyceae, Prymnesiophyceae and Chrysophyceae increased significantly in all areas. Cyanophyceae typically respond to increasing temperatures as they thrive in warm waters [6], and the N-fixing genera (such as *Aphanizomenon*, *Dolichospermum = Anabaena* and *Nodularia* spp.) are favoured by high P (and low N:P) levels. In addition, several species of Cyanophyceae originate from freshwater, and may thus be favoured by decreasing salinities [31].

Prymnesiophyceae (here mainly represented by *Chrysochromulina* spp.) are potentially toxic nanoflagellates, which can efficiently utilize the low nutrient levels typical during summer [32]. *Chrysochromulina* spp. biomass correlates positively with the N:P ratio, probably due to mixotrophy: they may be able to utilize phosphate from bacteria or picoplankton and thus achieve competitive advantage compared to fully autotrophic species in a P-limited environment [33]. In addition, phosphate deficiency promotes toxicity of this genus [34]. *Chrysochromulina* biomass also correlates with low summer surface water salinity [33]. In our study, Prymnesiophyceae were closely related to temperature in all three basins studied (Fig. 5). This suggests that a combination of changes in salinity, temperature and possibly N:P ratios [21] results in an environment that favours Prymnesiophyceae, even if significant trends in DIN:DIP ratios were not observed in the present study. The biomass increase of another nanoflagellate class, Chrysophyceae (mainly represented by *Pseudopedinella* spp.) seems connected to eutrophication, as shown in experimental studies [35].

Cryptophyceae was the only phytoplankton class that clearly decreased in all areas during the study period. Globally, Cryptophyceae occur during all seasons [36], but there are many studies implying that they are important especially in cooler waters [37], [38]. This agrees with our results showing that Cryptophyceae decreased with increasing warming.

Biodiversity of cool-water phyto- and zooplankton will decrease in the future Baltic Sea if temperatures continue rising at the current pace. On the other hand, based on our study harmful blooms formed by the N-fixing cyanobacteria and *Chrysochromulina* spp. can be expected to increase in magnitude, although another recent study shows that the occurrence frequency of cyanobacteria has not changed between years 1903–1911 and 1993–2005 [18]. In addition to Cyanophyceae and Prymnesiophyceae, also Dinophyceae that increased in the NBP [21], [39] include many potentially harmful species (e.g. *Dinophysis* spp., *Prorocentrum* spp., *Protoceratium reticulatum*). The detected phytoplankton community changes probably have direct effects on the food quality of micro- and mesozooplanktonic grazers, since Cryptophyceae are generally considered as high-quality food [40], whereas Cyanophyceae and Prymnesiophyceae seem to be low-quality food for herbivorous zooplankton [41], [42].

All five dominant mesozooplankton taxa (the cladocerans *Eubosmina, Evadne, Podon,* and the copepods *Acartia* and *Eurytemora* spp.) decreased in the GF, resulting in a decrease in total abundance of zooplankton (Fig. 3, Tables 2, 3). The reasons for the observed zooplankton decrease are not totally clear, but probably involve several factors such as warming of the water, decrease in salinity, shift in phytoplankton composition and altered predation pressure [43]. Together with the decrease in cladocerans, the most obvious change in the zooplankton community composition in the NBP was the increase of rotifers, whereas in the ÅS both the rotifer *Keratella* and the cladoceran *Eubosmina* increased. At least in the NBP the rotifer increase occurs in line with the salinity decrease [27]. Due to strong physiological constraints of saltwater on most rotifers, this group will be in main focus in future estuarine climate studies [27], as they constitute one of the first zooplankton groups to respond to climate-induced salinity changes. Apart from rotifers, juvenile cladocerans and copepodite stages, our monitoring data set does not provide deeper insights into potential changes in microzooplankton (20–200 µm). This limitation of the data set clearly demonstrates the need for expansion of existing monitoring and warrant further research on the role of microzooplankton in ecosystems exposed to climate change and eutrophication.

Phytoplankton and bacteria form the basis of the aquatic pelagic food web, supporting higher trophic levels through the classical food web or the microbial food web [44]. Increased temperature influences water column stratification that in turn affects vertical mixing processes, upward flux of nutrients, and effective light climate [45]. Although the data available did not allow analyses of long-term changes in stratification and mixed layer depth, such alterations to the environment have been shown to influence community composition and productivity in the northern Baltic Sea [46]. In addition, increasing river runoff and warming in combination with increased nutrient load can favour a bacterial-based food web [47]. In the current study, a decrease in “high-quality” phytoplankton food, together with an increase in mixotrophic and “low-quality” phytoplankton food for micro- and mesozooplankton, increase in small zooplankton such as rotifers, and a decrease in copepods can indicate a shift in the food web structure towards a more microbial, less energy-efficient food web. This could further imply that less energy reaches the grazing zooplankton and fish. In summary, a shift at the base of the food web, combined with stressors, such as warming (and thereby an increased metabolic demand by the grazers) could imply a severe disadvantage for mesozooplankton.

Apart from climate and eutrophication induced changes, the overexploitation of resources e.g., overfishing of cod in the Baltic Sea has resulted in a trophic cascade leading to reduced predation pressure on sprat (*Sprattus sprattus*) that, in turn, influence the summer phytoplankton community composition and biomass by heavily regulating the mesozooplankton community composition and abundance [8], [43]. This led Casini et al. [8] to suggest that the central Baltic summer plankton community is mainly governed by top-down processes, rather than via bottom-up regulation.

It is noteworthy that the decrease in zooplankton in the current study was mainly due to a decrease in the adult population, whereas juveniles/copepodites generally remained unaffected (Table 3). This can, in part, be explained by intense and selective feeding by sprat and Baltic herring (*Clupea harengus membras*), but also by a shift in food quality available for zooplankton, i.e., in terms of changes in phytoplankton species composition (Table 2; Figs. 2, 3, 4). However, as temperature is a major environmental factor influencing the study area, we suggest that in addition to a shift in phytoplankton composition and predation by fish, the observed pattern is in accordance with the population age-structure hypothesis. This hypothesis states that increased temperature increases the metabolism of younger and smaller age classes, giving them a competitive advantage over larger ones [12], [48], thus shifting the population towards younger and smaller sized age classes. This may, in turn, hamper the feeding conditions of planktivorous fish, which mainly select large copepods for food [49].

Marine ecosystems, such as the Baltic Sea are currently under strong anthropogenic pressure. Human-induced changes, such as climate warming, eutrophication and overexploitation of resources significantly affect ecosystem structure and function. The anthropogenically induced changes and natural processes do not act in isolation; rather the changes in plankton communities and ecosystems seem to be driven by complex interactions of several drivers. Plankton community responses to changes in the environment have commonly been assessed through small-scale laboratory studies with manipulation of a limited set of factors. Although such studies can highlight underlying mechanisms, implications for the ecosystem are hard to foresee. In this respect, long-term monitoring data are a valuable tool for documenting and understanding impacts of environmental change.

In conclusion, the present study shows that since 1979,

- Summer surface water temperature increased in all investigated sub-basins, whereas salinity decreased in the NBP.
- DIN and DIP concentrations increased in the GF, and DIN concentrations in the ÅS.
- Late summer chlorophyll *a* concentration and the biomass of Cyanophyceae, Prymnesiophyceae and Chrysophyceae increased, whereas the biomass of Cryptophyceae decreased in all basins.
- Total abundances of zooplankton, copepods and cladocerans decreased in the GF, but rotifers increased in the NBP and the ÅS.

Together, the observed changes in the Baltic Sea plankton communities suggest a shift in the food web structure towards more microbial, less energy-efficient food webs consisting of lower-food-quality and smaller sized organisms, which in combination with warming may lead to decreased availability of energy for grazing zooplankton and fish.
